# Effects of complex whole-body movements on EEG activity: a scoping review

**DOI:** 10.3389/fpsyg.2025.1547022

**Published:** 2025-07-31

**Authors:** Gabriel Müller, Atef Salem, Wolfgang I. Schöllhorn

**Affiliations:** Department of Training and Movement Science, Institute of Sport Science, Johannes Gutenberg-University, Mainz, Germany

**Keywords:** electroencephalography, exercise, physical activity, coordination, neural effects

## Abstract

**Introduction:**

To understand brain function, diverse approaches are pursued. The influence of movements on brain activity has been part of this research for decades. Recent advancements in electroencephalography (EEG) coupled with a shift in focus toward the effects of complex whole-body movements provided additional inspirations in this area. The investigation of the effects of endurance sports on brain activity poses the problem that an increase in EEG activity does not allow a differentiation between the effects caused by an increase in metabolism and those caused by an increase in the coordinative demands that must be coped with in parallel. This issue is currently being increasingly investigated using movements in which metabolism is not increased accordingly. This scoping review aims to summarize studies that investigated the acute effects of complex whole-body movements with increased parallel information processing on electrical brain activity to identify trends and potential issues that can be considered for future research.

**Methods:**

A comprehensive search across five scientific databases (PubMed, Web of Science, Scopus, SPORTDiscus, ProQuest) was conducted for studies that examined the acute effects of complex movements on EEG activity. The quality of the studies was assessed using a combination of the Quality Assessment Tool for Quantitative Studies (QATQS) and a modified quality assessment tool to evaluate EEG data acquisition and analysis.

**Results:**

Thirteen studies met the inclusion criteria for our scoping review, showing considerable heterogeneity in terms of design and type of movement. Nevertheless, the findings revealed a trend toward increased theta and alpha activity in frontal, central, and parietal areas during and after movement. In other frequency bands the findings were not consistent.

**Discussion:**

These findings are discussed in the context of possible moderating factors. Based on a consistent EEG methodology, future research should increasingly investigate the complexity of movements with regard to a clearer differentiation of cognitive demands to identify these as potential moderator variables.

## 1 Introduction

To understand brain function in the context of movement planning and execution, the acute effects of exercise on brain activity have garnered significant attention in recent decades. After an initial focus on fine motor sequential movements in psychology (Baddeley and Hitch, 1974), especially in the field of sport science the focus increasingly shifted to gross motor movements under the influence of primarily conditional parameters such as endurance and strength (Boutcher and Landers, 1988; Hosang et al., 2022; Hottenrott et al., 2013). Since in both cases the influence of the coordinative component is difficult to distinguish from the influence of the parallel increase in metabolism or voluntary effort, interest grew in the effects of movements on brain activity that are coordinatively more complex in terms of number of parallel activities while causing only minor changes in metabolism and less voluntary effort (Budde et al., 2008; Pesce, 2012). This shift was partly due to the observation of positive effects of exercise on executive functions (Benzing et al., 2016). In this context, electroencephalography (EEG) offered a promising, non-invasive approach with the potential to measure brain activity and distinguish different degrees of cognitive load depending on movement complexity (Sauseng et al., 2007). EEG signals are typically differentiated by delta (0.5–4 Hz), theta (4–8 Hz), alpha (8–13 Hz), beta (13–30 Hz), and gamma frequency bands (>30 Hz; Teplan, 2002). In some studies, a further distinction is made within the alpha and beta bands into alpha-1 (8–10.5 Hz), alpha-2 (10.5–13 Hz), beta-1 (13–15 Hz), and beta-2 (15–30 Hz) (Büchel et al., 2021; Hottenrott et al., 2013). These frequency bands are typically associated with certain behavioral phenomena in which these frequencies are commonly observed.

Delta waves are conventionally associated with deep sleep and have rarely been analyzed in the context of sport-related movements (Abhang et al., 2016). However, recent studies also suggested their involvement in cognitive processing (Harmony, 2013; Malik and Amin, 2017). Theta waves are typically observed during relaxed wakefulness and reduced vigilance (Mari-Acevedo et al., 2019; Zschocke et al., 2012). They have also been associated with memory processes (Herweg et al., 2020), the encoding of new information (Klimesch, 1999) and, in particular, characterized by frontal midline theta, with attention and executive control processes (Aftanas and Golocheikine, 2001; Cavanagh and Frank, 2014; Cavanagh and Shackman, 2015; Doppelmayr et al., 2008). Sauseng et al. (2007) found a dependence of anterior midline theta power on the level of mental effort during movements whose complexity was defined by the length of the key presses or path sequences. Alpha waves are the dominant waves in the normal waking state. They are also associated with attentional processes and play a role in various memory processes, such as working memory (Palva and Palva, 2007), semantic memory (Klimesch, 1997) and long-term memory (Klimesch, 1999). More recent theories suggest that alpha waves may be related to the active inhibition of task-irrelevant areas (Klimesch et al., 2007). A further differentiation assigns attention processes to the alpha-1 band and memory processes primarily to the alpha-2 band (Klimesch, 1999; Klimesch, 1997). Beta waves are associated with anxiety, problem solving, and deep concentration (Kirschbaum, 2008; Malik and Amin, 2017). Engel and Fries (2010) attribute beta waves to attention-related top-down processes and the sensorimotor system. From a more differentiated perspective, lower beta-1 waves are increasingly evident in focused concentration (Kirschbaum, 2008), while beta-2 waves are characteristic of increased arousal such as stress or excitement (Abhang et al., 2016). Gamma waves play an important role in attention as well as in working and long-term memory (Herrmann and Mecklinger, 2001; Jensen et al., 2007). Most recent studies have even detected higher frequencies spanning gamma/epsilon (60–150 Hz), ripple (80–250 Hz) and higher frequency ranges and suggest that these are involved in the encoding and retrieval of episodic memories and contribute to the formation and reactivation of memory traces (Kucewicz et al., 2024).

The interpretation of the frequency bands is based on an assignment of the individual frequency bands to certain activities, usually relying on correlations between activities with simultaneous derivation of brain activity. Since brain activity only reflects momentary behavioral activities and specific conditions, interpretations of the frequency bands are always limited to these and can hardly be generalized epistemologically. To date, the most common frequency band interpretations have been based almost exclusively on data collected in laboratory settings during stationary, seated or lying activities involving primarily cognitive tasks (Klimesch et al., 1998; Klimesch et al., 1997; Sauseng et al., 2007; Sauseng et al., 2006; Sauseng et al., 2005).

In recent decades, however, the influence of physical activity on brain oscillations has become increasingly important (Boutcher and Landers, 1988; Kubitz and Landers, 1993). Previous studies have primarily focused on endurance exercises such as running or cycling (for a review see Crabbe and Dishman, 2004; Gramkow et al., 2020; Hosang et al., 2022). These sports are characterized by a high level of automatization, typically requiring little conscious focus on movement execution but with a dominant metabolic component. In these studies, it is difficult to determine the influence of the coordinative requirements on brain activation. Recent advances in data preprocessing and technical developments, such as wireless EEG, enable measurements to be taken during movement (Mehta and Parasuraman, 2013), allowing for recordings during exercises that involve a greater range of motion, usually associated with increased coordination requirements and a reduced metabolic component. These developments combined with findings from behavioral psychology (Sibley and Etnier, 2003; Tomporowski, 2003) have shifted the focus toward a greater emphasis on the mental load induced by whole-body movements (Baumeister et al., 2008; Budde et al., 2008; Heilmann et al., 2022), which can vary depending on the amount of information that needs to be processed in parallel. The coordinative character of a movement, adaptations to a changing environment, or reactions to opponents and unpredictable situations can increase that kind of information. In the following, exercises that vary in these influencing factors are described as complex exercises. Here it is helpful to differentiate sequential complexity, where it is about the length and number of activities in a sequence, from parallel complexity, where it is about the number of activities performed in parallel. These factors appear to have varying effects on EEG activity, depending on the type of complexity of a movement. Even a slight increase in movement complexity seems capable of leading to neural adaptation. For example, increased modulation of efficiency in the alpha-2 network was observed after cross-country skiing, considered as a movement with increased parallel complexity, compared to conventional running in a laboratory environment. The increased coordinative demand in this case is associated with increased arm-leg coordination required in cross-country skiing compared to conventional running (Büchel et al., 2023). Previous reviews in the area of whole-body movements primarily analyzed running and cycling exercises with various metabolic loads in combination with EEG. Despite considerable heterogeneity in terms of measurement and processing of EEG data in the included studies, these reviews frequently reported increases in alpha and beta frequency bands. In contrast delta, theta, and gamma frequency bands showed inconsistent results (Gramkow et al., 2020; Hosang et al., 2022). Remarkably, these reviews neglected sports movements that require dominant cognitive engagement related to parallel movement complexity. Previous studies provide indications that the cognitive demand related to the parallel complexity of an exercise is reflected in EEG activity. In particular, a consistent increase in theta activity was observed during balance exercises (Gebel et al., 2020; Hülsdünker et al., 2015; Wittenberg et al., 2017) or target-shooting tasks in standing positions (e.g., rifle shooting; Doppelmayr et al., 2008). However, it remains unclear whether the increase in theta activity occurs across a broad range of complex gross motor movements, which could indicate a general increase in working memory and attentional processes (Sauseng et al., 2010; Sauseng et al., 2007), or whether it is limited to movement-specific requirements. Due to the growing number of studies investigating complex whole-body movements, a scoping review provides an overview of the effects of movement complexity on neural oscillations, focusing on movements that involve a greater range of motion than basic balance exercises. This may indicate trends as well as neglected areas that contribute to the discovery of further potential moderators in connection with EEG activity. Therefore, this scoping review aims to examine studies that analyze cortical activations in healthy individuals triggered by the acute execution of complex movements with various metabolic and coordinative demands.

## 2 Methods

### 2.1 Study protocol

A scoping review was conducted according to the guidelines of the Preferred Reporting Items for Systematic Reviews and Meta-Analyses (PRISMA) – extension for scoping review (Tricco et al., 2018).

### 2.2 Search strategy, selection process and eligibility criteria

A comprehensive search was conducted in five databases (PubMed, Web of Science, Scopus, SPORTDiscus and ProQuest) up to 06.06.2024. Appropriate Boolean operators (AND, OR and NOT) were used to join the various keywords. The following term was used for the search: (exercise OR “physical activity”) AND (EEG OR electroencephalography). Duplicate articles were removed using the EndNote software (version 20; The EndNote Team, 2013). The selection process was conducted independently by two authors, and any disagreement between the two authors was solved by consensus. The studies were assessed based on their title and abstract, followed by an analysis of the full text to determine whether they met the previously defined eligibility criteria. These criteria were: (1) studies had to be peer-reviewed and written in English, (2) the subjects had to be healthy participants, (3) the intervention had to consist of an acute physical exercise with increased mental demands (complex exercise), (4) studies had to provide a comparison over a certain measurement time or against another type of sport, (5) studies had to measure cortical activity using EEG.

**Figure 1:**
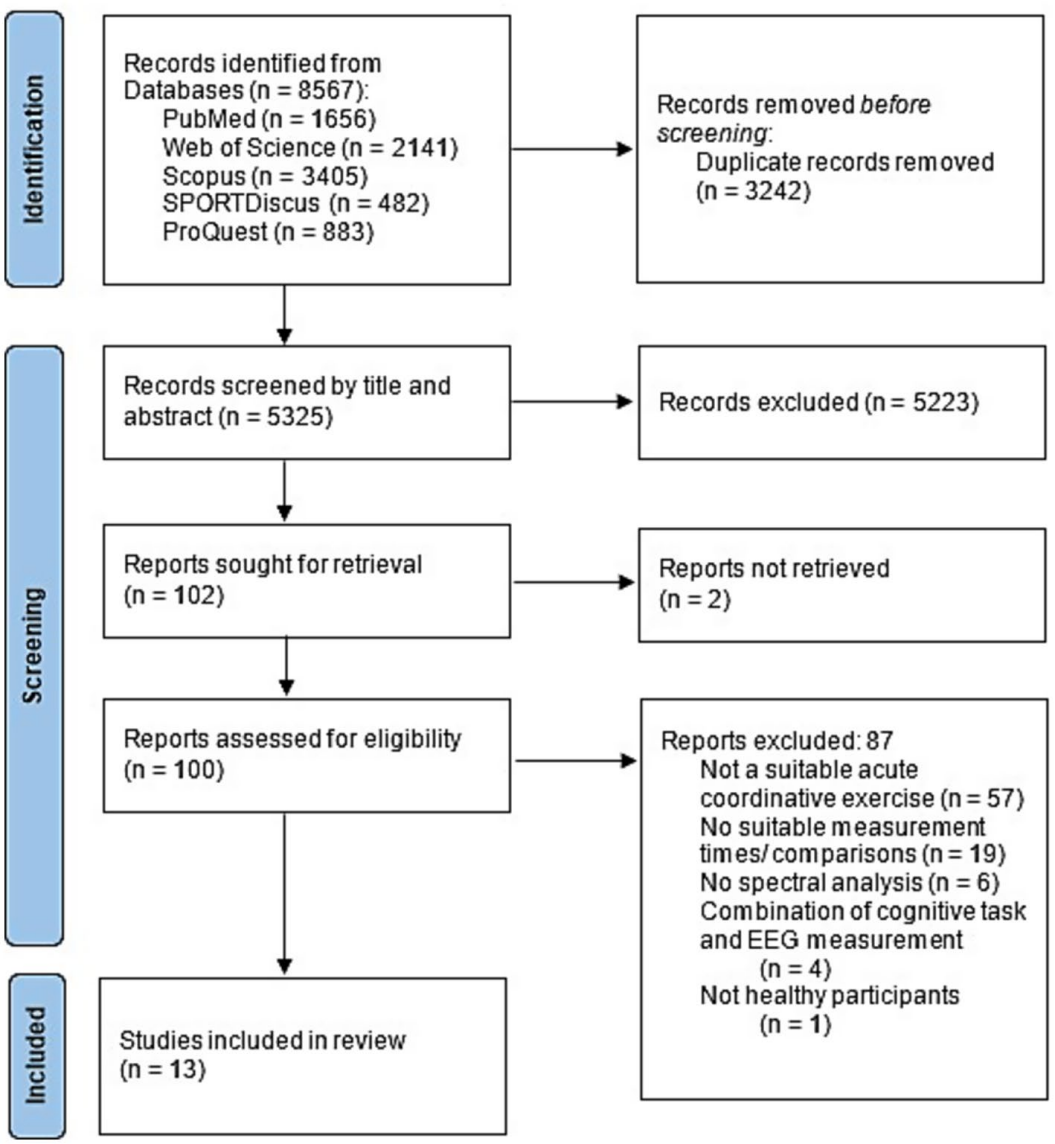
PRISMA flow diagram illustrating the literature search.

Studies were excluded if the exercise consisted of pure endurance (e.g., running, cycling) or strength exercise with a small number of degrees of freedom (e.g., bench press, squats). Pure endurance sports without any mental demand were excluded due to their low and monotonous coordinative demand. The aim was to focus mostly on the coordinative aspect. Event-related potentials and missing spectral analysis (Büchel et al., 2023; John et al., 2022) led to exclusion as well as studies in which cognitive tests were performed simultaneously with exercise (e.g., dual-task studies; Kahya et al., 2022), as the effects were only of interest triggered by the exercise. Published abstracts or conference papers were also excluded from further analysis. The screening process is shown in Figure 1. Both the search for suitable studies and the quality assessment were carried out independently by the first and second authors.

### 2.3 Data extraction

The author, year, number of participants, average age of participants, participant information, study design, type of exercise, intervention content, and intensity of exercise were extracted as important information from each eligible study. To provide an adequate overview of the EEG measurements, information on the analyzed frequency bands, duration of the EEG recording, number of electrodes, and the main results were also extracted. This information is presented in Tables 1–3.

**Table 1:** An overview showing design characteristics of the included studies.

Authors	Year	*N*	*M*_age_ (*SD*)	Participants	Design	Exercise	Intervention	EEG measurement
Ammar et al.^a^	2024	16	23.13 (2.09)	Healthy highly active males.	Within-subject-design.	Weightlifting (snatch).	Snatch (weightlifting) learning bout consisting of 36 trials using four different learning approaches: Repetitive learning (RL), Contextual interference (blocked, CIb; serial, CIs), and differential learning (DL). About 3 min bout duration. Mean HR (SD) after exercise: RL 85.23 (14.24); CIb 77.89 (12.29); CIs 77.85 (12.94); DL 84.47 (11.11).	Pre-post with eyes open for 5 min.
Baumeister et al.	2010	10	26.0 (0.7)	Healthy male golfers.	Within-subject-design.	Golf putting.	Putting for 3 min in a real and in a virtual Wii condition. HR: Not stated.	During-rest (after).
Becker et al.^b^	2023	20 (17 included into data analysis).	8.75 (0.91)	Healthy children.	Within-subject-design.	Movement game “crazy policemen” (exergame).	Movement exercise game, in which participants must follow the movement instructions of the policemen, with a low cognitive demand (LCD) and a high cognitive demand (HCD). Each game consists of 4 laps. Each lap lasts approximately 5 min. Low to moderate exercise intensity with 42% of maximal HR in both conditions.	Pre-during with eyes open for 3 min.
Ben-Soussan et al.^c^	2013	27	20–35 (range, SD = 2.5).	Healthy female students.	Between-subject-design.	Quadrato motor training, Simple motor training.	Quadrato motor training (QMT): Standing in a corner of a 0.5 × 0.5 m square participants moved to different corners in response to verbal instructions from an audio taper for 7 min. Simple motor training (SMT): Same manner as QMT, but with a fixed sequence. HR: Not stated.	Pre-post with eyes open/closed for 5 min (2.5 open, 2.5 closed).
Chu et al.	2018	12	21.7 (0.83).	Healthy male athletes.	Within-subject-design.	Taekwondo	Different Taekwondo kicks with either the left or the right foot. Each session lasted 3 min. HR: Not stated.	Pre-during.
Henz and Schöllhorn^d^	2016	24	25.3 (age range 18–34).	Healthy semiprofessional badminton players (males and females).	Within-subject-design.	Badminton serves.	Sixty badminton serves were executed using the Differential Learning (DL) approach or the Repetitive Learning (RL) approach (control group). HR: Not stated.	Pre-post with eyes open for 4 min.
Henz et al.^e^	2018	22	23.2 (age range 18–32).	Healthy adults.	Within-subject-design.	Badminton serves.	Badminton serves were executed for 20 min using four different motor learning approaches: Repetitive Learning (RL), Contextual Interference Learning (CI), gradual and chaotic Differential Learning (gr. DL; ch. DL). HR: Not stated.	Pre-post with eyes open for 4 min.
John and Schöllhorn^f^	2018	10	24.6 (3.31)	Healthy male adults.	Within-subject-design.	Rope-skipping	3 min rope skipping according to three motor learning approaches: Differential Learning without instruction (DLc), with instruction (DLi), and Repetitive Learning (RL). HR during exercise: DLc 154.6 (11.2); DLi 155.8 (11.3); RL 149.5 (15.0).	Pre-post with eyes open for 5 min pre and 6×5 minutes post exercise.
Rydzik et al.	2024	100	26.06 (4.9)	Healthy male professional kickboxers.	Between-subject-design.	Kickboxing	Simulation of sparring in the ring according to the K1 rules. The bout consisted of 3 rounds lasting 2 min each. High intensive exercise with a mean HR of 182.67 (5.9).	Pre-post with eyes open/closed for 6 min (3 open, 3 closed). Only data from eyes open analyzed.
Visser et al.^g^	2022	21 (18 included into data analysis).	24.72 (3.32)	Healthy adults.	Within-subject-design.	Table tennis	Playing cooperatively table tennis (TT) for 3 min. Other condition for 3 min on a cycling ergometer (EC). HR during exercise: TT 91.3 (14.0); EC 103.2 (15.2).	During with eyes open.
Wind et al.	2020	11	24.3 (2.45)	Healthy female adults.	Within-subject-design.	Dancing	Execution of 15 min Modern Jazz dance. HR: Not stated.	Pre-post with eyes open for 2 min.
Wollseiffen et al.^h^	2016	50 (10 included for the boxing intervention).	42 (10) women. 40 (12) men	Healthy male and female adults.	Between-subject-design.	Boxing	3 min of maximal effort boxing on a freestanding punching bag, including light and hard uppercuts, steps, jumps and direct punches. Other groups performed a biking exercise or no exercise. HR after boxing exercise: 166.8 (21.6).	Pre-post with eyes closed.
Wu et al.	2023	49 (25 novices, 24 experts).	Novices: 19.72 (1.08) Experts: 20.46 (0.93)	Healthy male adults.	Between-subject-design.	Dragon boat.	Execution of a 1,000 m exercise on a D1-M dragon boat dynamometer at full intensity. HR peak after completing: novices: 178.34 (6.01), experts: 170.37 (5.21).	Pre-post with eyes closed for 6 min.

*M*, mean; HR, heart rate; *SD*, standard deviation. The letters following certain authors^a–h^ represent studies included additional comparisons between different kinds of sports, different learning approaches within their research.

**Table 2:** An overview of the EEG-related information included the main findings from pre-post-measurement studies.

Authors	Year	Analyzed waves	Number of electrodes	Main finding
Ammar et al.	2024	Theta, Alpha, Beta, Gamma	19: Fp1, Fp2, F7, F3, F4, Fz, F8, T3, T4, T5, T6, C3, Cz, C4, P3, Pz, P4, O1, O2	Central (c), frontal (f), parietal/occipital (p/o), temporal (t), right temporal (r-t), left-temporal (l-t). RL: increased alpha in c, r-t and p/o regions; increased beta in c, r-t and p/o regions; increased gamma in f, c, r-t and p/o regions. CIb: increased alpha in c and p/o regions; increased beta in f, c, t and p/o regions; increased gamma in f, c, p/o and t regions. CIs: increased alpha in f and p/o regions; increased beta in f, c and p/o regions; increased gamma in f, c and p/o regions. DL: increased alpha in r-t and p/o regions; increased beta in c, r-t and p/o regions; increased gamma in c, r-t and p/o regions. Alpha: CIs lower increase than RL in r-t region; beta: CIs lower increase than CIb in r-t region; gamma: DL lower increase than CIb in the l-t region.
Ben-Soussan et al.	2013	Alpha	6: F3, F4, T7, T8, P5, P6	QMT: no change in the alpha power. SMT: no change in the alpha power. No difference in alpha power between QMT and SMT.
Henz and Schöllhorn	2016	Theta, Alpha, Beta, Gamma	19: Fp1, Fp2, F7, F3, F4, Fz, F8, T3, C3, Cz, C4, T4, T5, P3, Pz, P4, T6, O1, O2	Increased DL theta power at central, parietal and occipital regions compared to baseline. Increased DL theta at central, parietal and occipital regions compared to RL. Increased DL alpha power compared to RL in central, parietal and occipital regions.
Henz et al.	2018	Theta, Alpha, Beta, Gamma	19: Fp1, Fp2, F7, F3, F4, Fz, F8, T3, T4, T5, T6, C3, Cz, C4, P3, Pz, P4, O1, O2	Increased theta in ch. DL/gr. DL compared to CI, RL and baseline rest at parietal electrodes. Increased theta in ch. DL compared to CI, RL, gradual DL and baseline rest at central electrodes. Increased alpha in RL compared to CI, gr. DL, ch. DL and baseline rest in occipital electrodes. Increased alpha in ch. DL compared to RL, CI, gradual DL and baseline rest at central electrodes. Increased alpha in ch. DL compared to CI, RL and baseline at parietal electrodes. Increased gamma in CI compared to gr. DL, ch. DL and RL at frontal electrodes. Comparisons: Increased gamma in CI compared to gr. DL, ch. DL, RL and baseline rest at central electrodes.
John and Schöllhorn	2018	Theta, alpha-1, alpha-2, beta-1, beta-2, beta-3, gamma	19: Fp1, Fp2, F7, F3, F4, Fz, F8, T3, T4, T5, T6, C3, Cz, C4, P3, Pz, P4, O1, O2	No changes from pre-to immediately post exercise in any of learning approaches. 5 min after exercise: Higher alpha-2 power in left temporal region in RL group in comparison with DLi.
Rydzik et al.	2024	Delta, theta, alpha, SMR, beta-1, beta-2	9: Fz, F3, F4, Cz, C3, C4, Pz, P3, P4	Theta increase in the frontal region, alpha increase in the parietal region, beta-1 increase in the frontal and parietal region.
Wind et al.	2020	Theta, alpha, beta, gamma	19: Fp1, Fp2, F7, F3, F4, Fz, F8, T3, T4, T5, T6, C3, Cz, C4, P3, Pz, P4, O1, O2	No changes in theta activity, alpha activity increase in frontal, central, right-temporal and parietal region, beta activity increase in right-and left-temporal region and decreased gamma activity in occipital region.
Wollseiffen et al.	2016	Alpha-1, alpha-2, beta-1, beta-2	3: Fp1, Fz, Fp2	No relative increase in alpha-1 activity, increase of frontal alpha-2 activity, no relative increase in beta-1 or beta-2 activity. No difference between boxing and cycling.
Wu et al.	2023 (Novice group)	Delta, theta, alpha-1, alpha-2, beta-1, beta-2	12: F3, Fz, F4, C3, Cz, C4, P3, Pz, P4, O1, Oz, O2	Higher delta in central region, higher theta in central region, higher alpha-1 in frontal and parietal region, higher beta-1 and beta-2 in the parietal region after exercise than pre-exercise.
	2023 (Expert group)	Delta, theta, alpha-1, alpha-2, beta-1, beta-2	12: F3, Fz, F4, C3, Cz, C4, P3, Pz, P4, O1, Oz, O2	Lower delta in frontal, central and occipital region, lower theta in frontal region, higher alpha-1 in occipital region, lower beta-1 in occipital region and higher beta-2 in the parietal and occipital region after exercise than pre-exercise.

RL, repetitive learning; CI, contextual interference; CIs, contextual interference serial; CIb, contextual interference blocked; DL, differential learning; ch. DL, chaotic differential learning; gr. DL, gradual differential learning; DLi, instructed differential learning; QMT, quadrato motor training; SMT, simple motor training.

**Table 3:** An overview of the EEG-related information included the main findings from during-measurement studies.

Authors	Year	Analyzed waves	Number of electrodes	Main finding
Baumeister et al.	2010 (Real environment)	Theta, alpha-1, alpha-2	6: Fz, F3, F4, Pz, P3, P4	Higher theta in the frontal region, no change in alpha-1activity and no change in alpha-2 activity during the exercise compared to rest.
	2010 (Virtual environment)	Theta, alpha-1, alpha-2	6: Fz, F3, F4, Pz, P3, P4	Higher theta activity in the frontal region, no change in alpha-1 activity and a higher alpha-2 activity in the parietal region during exercise compared to rest.
Becker et al.	2023	Theta, alpha-1, alpha-2	64 Channels	LCD-condition: increased theta and alpha-1 activity in the prefrontal region, decreased alpha-1 in left and right motor, left and right parieto-occipital and occipital region, decreased alpha-2 activity in left motor and left parieto-occipital regions during vs. baseline. HCD-condition: increased theta and alpha-1 in the prefrontal region, decreased alpha-1 in left motor, left and right parieto-occipital and occipital regions, decreased alpha-2 activity in the left motor region. LCD vs. HCD: higher theta in prefrontal region and lower alpha-1 in occipital region in HCD during exercise.
Chu et al.	2018	Delta, theta, alpha, beta, gamma	One electrode on the left forehead	Decrease of gamma activity in frontal region with both the left and right foot during exercise compared to before.
Visser et al.	2022	Theta	64 Channels	Higher theta power in TT compared to EC in the prefrontal and frontocentral region during the exercise.

LCD, lower cognitive demand; HCD, higher cognitive demand; TT, table tennis; EC, ergometer cycling.

### 2.4 Quality assessment

The quality of the studies was assessed using a combination of the Quality Assessment Tool for Quantitative Studies (QATQS; National Collaborating Centre for Methods and Tools, 2008) and a modified^1^ quality assessment tool to evaluate EEG data acquisition and analysis based on Parr et al. (2021). QATQS includes six criteria: (1) selection bias, (2) study design, (3) confounders, (4) blinding, (5) data collection method and (6) withdrawals and dropouts. It is used to assess the overall study quality. The modified quality assessment tool by Parr et al. (2021) includes the criteria (a) artifact handling, (b) brain wave definition,^2^ (c) regional specificity, (d) temporal precision, and (e) controls to volume conduction. This tool is used to assess the quality of EEG measurements. Each of these 11 criteria was given a quality score from 1 to 3 (1 = strong quality; 2 = moderate quality; 3 = weak quality). As the assessment tool of Parr et al. (2021) is used to assess the EEG measurement, this output is used to assess criterion (5) data collection method in the QATQS. Consequently, in the modified tool by Parr et al. (2021), no weak assessment in criteria a – e results in a score of 1 (strong quality) for the criterion data collection method, one weak rating results in a score of 2 (moderate quality) and more than one weak rating results in a score of 3 (weak quality). Overall, a study is assigned the value 1 if it does not show the value “weak quality” in any of the criteria (1–6), a 2 if it shows the value once and a 3 if more than one criterion is rated as weak. A detailed list of the quality assessments is provided in Supplementary Table S1.

## 3 Results

The initial search in five databases yielded 8,567 records. A total of 3,242 articles were removed as duplicates. 5,325 articles were screened by title and abstract, of which 5,223 studies were excluded by title and abstract. Three studies could not be retrieved. One of the three was successfully obtained from the author upon request. After careful review of 100 full-text articles, 13 articles met the eligibility criteria and were included in our scoping review. Further studies were excluded for the following reasons: studies whose exercise was not acute and coordinative according to the above mentioned characteristics (*n* = 57), no suitable measurement time points or comparisons were made (*n* = 19), no spectral analysis was conducted (*n* = 6), a cognitive task was performed simultaneously with EEG measurement (*n* = 4), or the study did not involve healthy participants (*n* = 1). This resulted in 13 studies that were included in this review. These can be divided into two groups: Studies that focused on EEG comparison before and after exercise (pre-post, *n* = 9) and studies that focused on measurement during exercise (during, *n* = 4).

### 3.1 Complex whole-body exercises

All selected studies (Tables 1–3) involved an acute complex exercise with healthy participants as the intervention. In 12 of the 13 studies, the subjects were adults, while one study examined children. In terms of content, the sports intervention exercises can be classified as follows: racket sports: badminton (Henz et al., 2018; Henz and Schöllhorn, 2016), golf (Baumeister et al., 2010) and table tennis (Visser et al., 2022); in the area of coordinative demands combined with force-velocity requirements: kickboxing (Rydzik et al., 2024), Taekwondo (Chu et al., 2018), boxing (Wollseiffen et al., 2016) and weightlifting (Ammar et al., 2024); in the area of coordinative demands combined with some endurance requirements: Quadrato Motor Training (QMT, Ben-Soussan et al., 2013), rope skipping (John and Schöllhorn, 2018), dragon boat (Wu et al., 2023); in exergames (“crazy policemen,” with its two variations lower cognitive demand (LCD) and higher cognitive demand (HCD); Becker et al., 2023); and dance (Wind et al., 2020). In four studies, the movements were executed within the framework of various learning models, including repetition learning (RL), contextual interference learning (CI) with its modifications serial (CIs) and blocked (CIb), and differential learning (DL) with its modifications: gradual (gr. DL), chaotic (ch. DL), instructed (DLi), and non-instructed (DLc) differential learning. The physical exertion varied between the studies and ranged from low metabolic exertion (putting; Baumeister et al., 2010) to maximum metabolic intensity (dragon boat; Wu et al., 2023). This was measured using heart rate (*n* = 7), while in some studies, no information was provided regarding the exercise-related metabolic intensity (*n* = 6).

### 3.2 Studies which compared EEG pre - and post-activity

#### 3.2.1 Delta activity

Delta activity was investigated in only two of the nine studies. Wu et al. (2023) observed an increase in delta activity in the central cortex of the novice group in the context of dragon boat racing, but a decrease in the frontal, central, and occipital regions of the expert group. However, Rydzik et al. (2024) found no changes in delta activity after kickboxing.

#### 3.2.2 Theta activity

Of the nine studies that measured the effects of exercise on brain activity in a pre-post-test design, seven analyzed the theta band. Four showed increases in the frontal region (*n* = 1; kickboxing, Rydzik et al., 2024), central region (*n* = 3; e.g. dragon boat (novice), Wu et al., 2023), in the occipital region for badminton serves (*n* = 1; Henz and Schöllhorn, 2016), and in the parietal region for the same movement (*n* = 2; ch. DL, gr. DL, Henz et al., 2018; Henz and Schöllhorn, 2016). Three studies found no changes (e.g., dance, Wind et al., 2020), while one study reported a decrease in theta activity in the frontal region in the context of a dragon boat race (expert, Wu et al., 2023).

#### 3.2.3 Alpha activity

All nine studies investigated changes in alpha activity. Six showed an increase after exercise compared to before. A further examination of the cortical areas revealed increases in the frontal (*n* = 4; e.g. dance, Wind et al., 2020), central (*n* = 3; e.g. badminton (ch. DL), Henz et al., 2018), parietal (*n* = 4; e.g. dragon boat (novice), Wu et al., 2023), occipital (*n* = 2; RL, Henz et al., 2018; experts, Wu et al., 2023), right temporal (*n* = 2; RL, DL, Ammar et al., 2024; Wind et al., 2020), and in the parietal/occipital cortex in the context of the snatch (*n* = 1; Ammar et al., 2024). Wollseiffen et al. (2016) observed an increase in alpha activity only in the alpha-2 band after boxing, whereas Wu et al. (2023) reported an enhancement only in the alpha-1 band in both groups of the dragon boat. Three studies (e.g., QMT, Ben-Soussan et al., 2013) reported no changes in the examined cortical areas after the exercise, and none of the studies found a decrease in alpha activity.

#### 3.2.4 Beta activity

Eight of the nine studies analyzed changes in beta activity from pre- to post-activity. Four reported an increase in beta activity. These increases were observed in the frontal (*n* = 2; snatch (CIs, CIb), Ammar et al., 2024; kickboxing, Rydzik et al., 2024), parietal (*n* = 2; Rydzik et al., 2024; dragon boat, Wu et al., 2023), parietal/occipital (*n* = 1; Ammar et al., 2024), central (*n* = 1; Ammar et al., 2024), left temporal (*n* = 2; CIb, weightlifting; Ammar et al., 2024; dance, Wind et al., 2020), right temporal (*n* = 2; RL, DL, CIb, weightlifting, Ammar et al., 2024; Wind et al., 2020), and occipital region (*n* = 1; dragon boat, experts, Wu et al., 2023). Rydzik et al. (2024) found an enhancement in the parietal and frontal cortex only in the beta-1 band after kickboxing, and Wu et al. (2023) observed the parietal and occipital increase after dragon boat racing in the expert group only in the beta-2 band. Four studies found no changes in beta activity in the examined areas (*n* = 4; e.g. rope skipping, John and Schöllhorn, 2018). Only one study (Wu et al., 2023) reported a decrease in beta-1 activity in the occipital cortex of the expert group in the context of dragon boat racing.

#### 3.2.5 Gamma activity

Five studies investigated gamma activity. Two reported an increase in gamma activity. This increase was observed in the frontal (*n* = 1; snatch (RL, CIs, CIb), Ammar et al., 2024), central (*n* = 2; Ammar et al., 2024; badminton (CI), Henz et al., 2018), right temporal (*n* = 1; RL, DL, CIb, Ammar et al., 2024), left temporal (*n* = 1; CIb, Ammar et al., 2024), and parietal/occipital region (*n* = 1; Ammar et al., 2024). Henz and Schöllhorn (2016) and John and Schöllhorn (2018) reported no change in gamma activity after badminton serves or rope skipping, and one study showed a decrease in gamma activity in the occipital region (dance, Wind et al., 2020).

The overall quality of the studies that measured before and after exercise was rated as moderate (*M* = 2.33).

### 3.3 Studies which measured EEG during the activity

#### 3.3.1 Delta activity

Delta activity was also investigated in only one study (Chu et al., 2018). This study showed no change in activity during exercise compared to before.

#### 3.3.2 Theta activity

Of the four studies that measured theta activity during movement, all of them investigated theta activity. Three of the four studies reported an increase during exercise. This increase was observed in the prefrontal cortex during the exergame and table tennis (*n* = 2; LCD, HCD, Becker et al., 2023; Visser et al., 2022), in the frontal cortex during golf putting compared to afterward (*n* = 1; Baumeister et al., 2010), and in the frontocentral region (*n* = 1; table tennis, Visser et al., 2022). Chu et al. (2018) reported no change in theta activity during Taekwondo compared to the resting state, and none of the studies showed a decrease.

#### 3.3.3 Alpha activity

Alpha activity was analyzed in three studies. Increases were reported in two investigations, namely during golf putting and the exergame (*n* = 2; Baumeister et al., 2010; Becker et al., 2023). These increases were observed as enhanced alpha-1 power in the prefrontal region (*n* = 1; exergame (LCD, HCD), Becker et al., 2023) and enhanced alpha-2 activity in the parietal region (*n* = 1; virtual golf putting, Baumeister et al., 2010). Chu et al. (2018) reported no changes compared to the resting state in alpha activity during Taekwondo movements. One study found a decrease in alpha activity (Becker et al., 2023), specifically a decrease in alpha-1 power in the left and right parieto-occipital regions (LCD, HCD), occipital region (LCD, HCD), left motor cortex (LCD, HCD), and right motor cortex (LCD) during the exergame compared to before. Additionally, it showed a reduction in alpha-2 power in the left parieto-occipital region (LCD) and left motor cortex (LCD, HCD).

#### 3.3.4 Beta activity

One study investigated beta activity during exercise (Chu et al., 2018). The authors reported no change in beta activity compared to before.

#### 3.3.5 Gamma activity

Gamma activity was also investigated in only one study (Taekwondo, Chu et al., 2018). This study showed a decrease in gamma activity in the frontal region.

The overall study quality in this area was rated as moderate (*M* = 2.5).

### 3.4 Studies comparing exercises

In eight^a–h^ of the 13 studies, a further comparison was made within the study between exercises that differed in their coordinative demands from the intervention exercise (see letters in Table 1). This comparison was made in two studies on cycling (table tennis, Visser et al., 2022; boxing, Wollseiffen et al., 2016), in four studies the exercise was performed using different learning approaches with different levels of variability (snatch, Ammar et al., 2024; badminton serve, Henz et al., 2018; badminton serve, Henz and Schöllhorn, 2016; rope skipping, John and Schöllhorn, 2018), and in two studies, the movement was varied by externally specifying the direction of movement and thus requiring a situational response with increased spatial orientation (exergame, Becker et al., 2023; QMT, Ben-Soussan et al., 2013). Wollseiffen et al. (2016) and Ammar et al. (2024) compared the increases from pre to post, while the others focused on the differences in the absolute EEG power between the movements at the respective measurement time. This was shown, for example, by Becker et al. (2023) by comparing LCD and HDC activity during movement and by Henz et al. (2018) by comparing EEG activity of different learning approaches after the intervention.

#### 3.4.1 Delta activity

None of the studies listed here examined delta activity.

#### 3.4.2 Theta activity

Of the eight studies that investigated differences in frequencies between exercises, six^abdefg^ studies investigated theta activity. Four reported differences in theta activity between exercises. This was reflected in an increase in the prefrontal (*n* = 2; HCD,^3^ Becker et al., 2023; TT, Visser et al., 2022), frontocentral (*n* = 1; TT, Visser et al., 2022), central (*n* = 2; chaotic DL, Henz et al., 2018; DL, Henz and Schöllhorn, 2016), parietal (*n* = 2; chaotic DL, Henz et al., 2018; DL, Henz and Schöllhorn, 2016), and occipital cortex (*n* = 1; DL, Henz and Schöllhorn, 2016). Two studies that executed the movements of snatching and rope skipping using different movement learning models found no differences in theta activity between the movement sequences (Ammar et al., 2024; John and Schöllhorn, 2018).

#### 3.4.3 Alpha activity

Alpha activity was investigated in seven^abcdefh^ of the eight studies. A difference in alpha activity was reported in five studies. This was found in the right temporal (*n* = 1; snatch (RL), Ammar et al., 2024), left temporal (*n* = 1; rope skipping (RL), John and Schöllhorn, 2018), occipital (*n* = 3; e.g. LCD, Becker et al., 2023), parietal (*n* = 2; e.g. chaotic DL, Henz et al., 2018), and central region (*n* = 2; chaotic DL, Henz et al., 2018; DL, Henz and Schöllhorn, 2016). The difference reported by Becker et al. (2023) in the occipital region was only shown in alpha-1 activity, and the difference in the left temporal region reported by John and Schöllhorn (2018) was only shown in alpha-2 activity. Two studies showed no difference in alpha activity between exercises (Ben-Soussan et al., 2013; Wollseiffen et al., 2016).

#### 3.4.4 Beta activity

Beta activity was examined in only five^adefh^ of the eight studies. Only Ammar et al. (2024) showed a greater increase in the group with a blocked sequence of exercise (contextual interference blocked) compared to the contextual interference serial group in the right temporal cortex. Four of the remaining studies showed no difference between the groups (*n* = 4; e.g. Wollseiffen et al., 2016) during or after the intervention.

#### 3.4.5 Gamma activity

Four^adef^ of the eight studies investigated gamma activity. Two studies showed differences between the exercises. These were found in the left temporal (*n* = 1; CIb, Ammar et al., 2024), frontal (*n* = 1; CI, Henz et al., 2018), and central cortex (*n* = 1; CI, Henz et al., 2018). Two studies reported no differences in gamma activity (Henz and Schöllhorn, 2016; John and Schöllhorn, 2018).

The overall quality of the studies, which included several exercises with different coordinative requirements, was rated as moderate (*M* = 2.25).

## 4 Discussion

The aim of this review was to provide an overview of the neural effects of acute, mentally demanding physical exercises. This demand is typically found in complex movements involving many degrees of freedom in parallel. The neural effects were investigated by spectral EEG activity either in a pre-post-test design or in a during design. In some of the studies, additional comparisons were made between exercises with varying coordinative demands. After the selection process, there were 13 studies left (pre-post = 9; during = 4). In 12 of the 13 studies, the participants were healthy adults and in one study, healthy children participated. The duration of the physical activities ranged from 3 to 20 min. Both cyclical (e.g., Wu et al., 2023) and non-cyclical (e.g., Henz and Schöllhorn, 2016) movements were analyzed.

### 4.1 Effects on delta frequency band

Delta oscillations are the least studied frequencies and were only analyzed in three studies (pre-post design = 2; during-design = 1). Wu et al. (2023) found an increase in delta activity in the beginner group and a decrease in the experienced group. No changes were found in two studies (Chu et al., 2018; Rydzik et al., 2024). These differences may be the result of the different performance levels of the respective groups. Lardon and Polich (1996) demonstrated differences in resting EEG delta activity between individuals who engage in regular physical activity and those who are less active. This, combined with the fact that delta activity is also associated with cognitive processing (Harmony, 2013), could explain the differences in this frequency band, as postural control in dragon boat racing is thought to require greater cognitive engagement in beginners. However, because of the small number of studies that included delta wave analysis it seems very speculative to assign any of these frequencies to sport-specific movements.

### 4.2 Effects on theta frequency band

**Figure 2:**
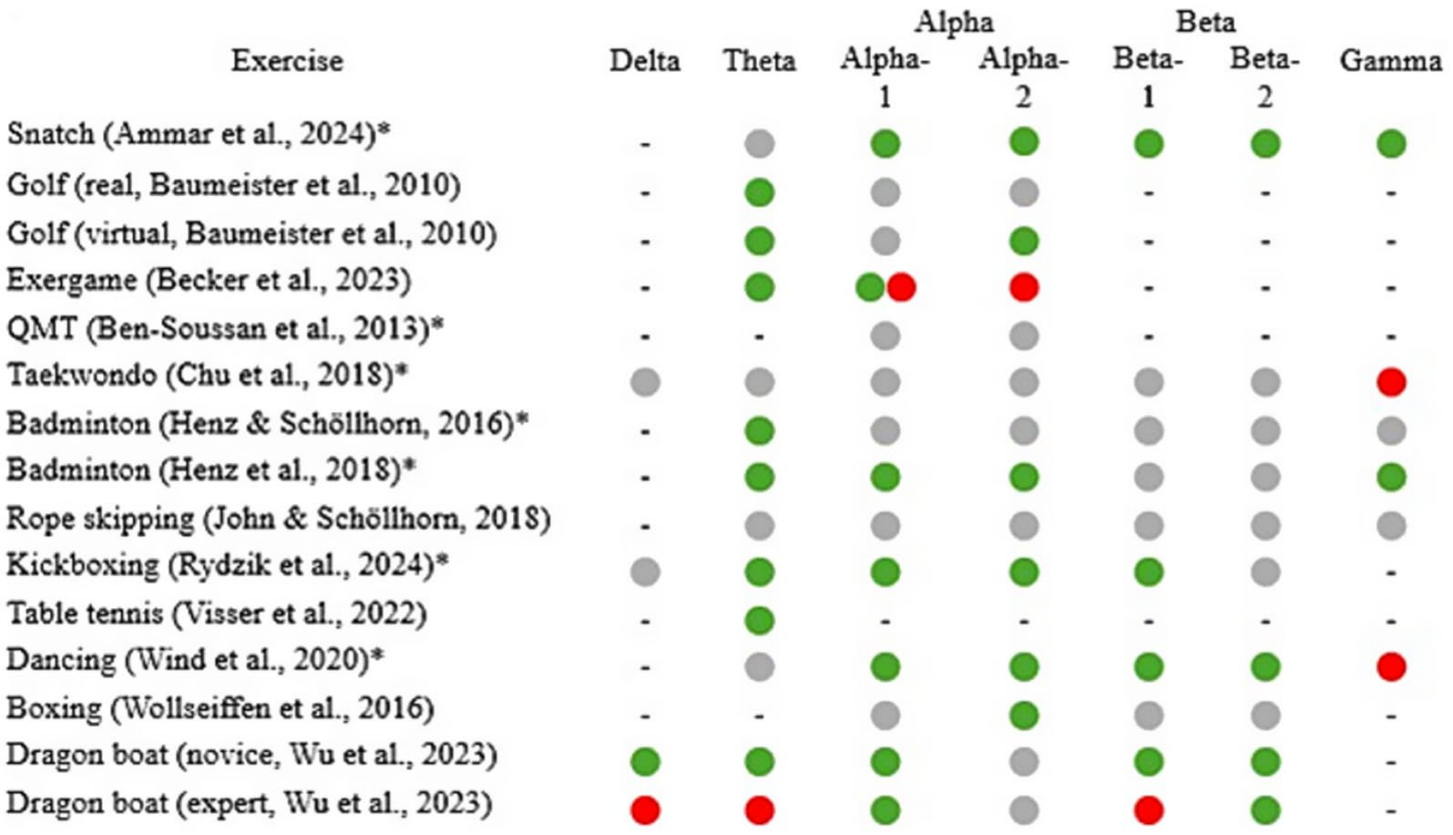
Overview of frequency-band specific EEG results from pre-post measurements and pre/after-during measurements. Green indicates an increase, red a decrease, a combination of both an increase and a decrease, gray indicates no change. A bar (−) indicates that the frequency was not analyzed. An asterisk after the author indicates that no distinction was made between alpha-1 and alpha-2 or beta-1 and beta-2 (Rydzik et al. (2024) only distinguished between beta-1 und beta-2). QMT, Quadrato motor training.

Seven studies (Figure 2) reported an increase in theta frequency (pre-post design = 4; during-design = 3). Three studies showed no change, and one reported a decrease. Increases were found in the central (*n* = 3; e.g. Henz and Schöllhorn, 2016), frontal (*n* = 2; Baumeister et al., 2010; Rydzik et al., 2024), prefrontal (*n* = 2; Becker et al., 2023; Visser et al., 2022), and frontocentral regions (*n* = 1; Visser et al., 2022). Theta activity in primary frontal areas is linked to cognitive tasks requiring increased executive control (Cavanagh and Frank, 2014; Cavanagh and Shackman, 2015; Jensen and Tesche, 2002) and attention (Aftanas and Golocheikine, 2001; Gevins, 1997). Similar findings have been observed in sports-related exercises. For example, increased frontal midline theta values were found in targeting exercises in the pre-shooting phase or balance exercises that required an increased level of executive control (Doppelmayr et al., 2008; Hülsdünker et al., 2016; Sipp et al., 2013). However, these increases could not be shown for cyclical, mostly automated movements with less parallel coordinative requirements (e.g., cycling, running, Gramkow et al., 2020; Hosang et al., 2022). More specifically, all studies that examined movements from the area of racket sports (*n* = 4; Baumeister et al., 2010; Henz et al., 2018; Henz and Schöllhorn, 2016; Visser et al., 2022) showed an increase in theta frequency. These movements were characterized by their goal-directed nature, specifically to strike the ball toward defined target areas. These movement-specific requirements may have contributed to this, which are not only characterized by higher demands on coordination and attention processes, but, e.g., in the case of table tennis, also by the additional situational reactions to the opponent, which increase the information to be processed by the visual information input and to which the corresponding reactions must be adapted (Visser et al., 2022). This visual information means that information from the visual cortex must be integrated additionally into the whole-body sensory and motoric activities. The synchronization of areas that are more distant tends to be attributed to lower frequencies such as theta frequencies (Varela et al., 2001; Fries, 2005). Since similar effects were found in balance exercises (Gebel et al., 2020; Hülsdünker et al., 2015), for example, in which multiple joints as well as visual and vestibular information have to be integrated in parallel to keep the center of gravity within the support area, common movement requirements such as increased cognitive control, especially in frontal areas (Cavanagh and Frank, 2014), could be responsible. An increase in theta activity was also observed after kickboxing (Rydzik et al., 2024), suggesting that other sport-specific demands may be responsible for this.

When considering theta frequency band and only the type of sports in general, the remaining categories of the examined complex exercises show inconsistent results and give reason to further investigate the movement-specific demands. One reason why not all studies showed an increase could lie in the factors influencing EEG activity, as reported in the study by Wu et al. (2023), which identified higher theta activity in novices than in experts in the context of dragon boat racing. This may indicate that with increasing experience, movements become more automated, thereby requiring fewer attentional resources, whereas novices may have increased demands on posture control and coordination. John and Schöllhorn (2018) also examined a cyclical movement. They did not find theta changes after rope skipping. It remains to be determined whether it is the difference between cyclical and non-cyclical movements or the individual’s level of expertise that plays a decisive role in this context. This issue requires further investigation.

Some of the studies carried out further analysis by comparing movements of different mental demands. They examined whether neural activity differed between the movements. Four^bdeg^ of the six studies that examined theta reported higher values in favor of movements with higher cognitive demand. Visser et al. (2022) showed this by comparing a table tennis exercise with an exercise on a bicycle ergometer, and Henz and Schöllhorn (2016) performed badminton serves using different learning approaches with increasing variations within the movement. One possible reason for this may be that frontal theta is related to the amount of mental effort (Sauseng et al., 2007). However, these results were not observed in all comparisons.

Four studies found no theta changes triggered by exercise (weightlifting, Ammar et al., 2024; Taekwondo, Chu et al., 2018; rope skipping, John and Schöllhorn, 2018; dancing, Wind et al., 2020). One of the reasons for the differences in the results could be the different demands of the movement on cognition or somatosensory execution. In all four studies, a movement with high parallel complexity is the object of investigation, but all four studies include additional factors that point to an excessive expression of an additional condition that indicates a shift in the ratio of dominance in the brain activities. In weightlifting, the voluntary effort to lift a barbell is added to the complex coordination (Ammar et al., 2024). The same applies to the foot kick in Taekwondo performed at maximum speed (Chu et al., 2018). Varying the jumping techniques with each jump during rope skipping also places excessive demands on creativity under time pressure (John and Schöllhorn, 2018), and performing a specific sequence of movements in dance (Wind et al., 2020) is also associated with increased demands on sequential memory and spatial planning. Ermutlu et al. (2015) found differences in resting theta activity between fast ball sports athletes and dancers, indicating the divergent effect of sport types on neural activity. Additionally, the different demands between cyclical and non-cyclical movements or an interaction effect between metabolic load and mental demand on theta activity cannot be excluded at this point either and requires further investigation.

Overall, many studies showed theta increases in frontal and central areas. This differs from previous reviews (Crabbe and Dishman, 2004; Gramkow et al., 2020; Hosang et al., 2022), which reported inconsistent results with regard to theta activity in endurance-dominated, mostly automated whole-body exercises. The results presented here reflect a trend that aligns with findings from the field of shooting (Doppelmayr et al., 2008) and balance (Hülsdünker et al., 2016) and expand the knowledge that similar effects on theta activity can also be found in exercises with increased range of motion, duration and intensity, in which the influence of various fatigue mechanisms additionally comes into play. Although theta activity in cognitive tasks is associated with the level of mental effort, it is necessary to take a more differentiated look at the specific cognitive demands of the individual movements, as these can vary significantly. Here rather more differentiation is recommended instead of hasty generalizations.

### 4.3 Effects on alpha frequency band

Eight studies reported an increase in alpha activity (pre-post-design = 6; during-design = 2), four studies showed no changes, and one study reported a decrease. The increases were primarily observed in frontal (*n* = 5; e.g. Wollseiffen et al., 2016), central (*n* = 3; e.g. Wind et al., 2020), and parietal regions (*n* = 6; e.g. Rydzik et al., 2024). Our results are partially consistent with previous research, which also found alpha increases during and after endurance-dominated exercises in frontal and central areas of the brain (Hosang et al., 2022). Hosang et al. (2022) attributed this to the inclusion of cognitive functions during and after exercise. Frontal alpha synchrony is associated with top-down control and inhibition processes when measured during activities (Klimesch et al., 2007; Misselhorn et al., 2019) and thus offers an explanation for the increase in these areas. However, the approach does not adequately explain the increase in alpha power with eyes closed or the fact that children under the age of 5 exhibit alpha as their highest frequency. The increased alpha activity in the parietal cortex differs from previous research findings. Parietal alpha activity is typically associated with spatial orientation (van Schouwenburg et al., 2017), internal attention (Benedek et al., 2014) and intersensory reorientation (Misselhorn et al., 2019). All three associations are typically considered as a necessity for whole-body athletic movements.

The simultaneous involvement of frontal and parietal areas is consistent with the previous hypothesis that the frontoparietal network is involved in the top-down control of attention (Corbetta and Shulman, 2002; Noudoost et al., 2010). While frontal lobe activity is mainly associated with top-down control, parietal activity is rather assigned to bottom-up control (Buschman and Miller, 2007). Regarding the increased demands on various attentional processes to successfully execute the exercises investigated here, this may explain the increased parietal alpha activity. Of the reported increases in alpha activity, two studies (Becker et al., 2023; Wu et al., 2023) only reported changes in alpha-1 frequency and two (Baumeister et al., 2010; Wollseiffen et al., 2016) only showed these in alpha-2 activity. Given that alpha activity has been shown to regularly increase during and after activities with enhanced metabolic demands (Gramkow et al., 2020; Hosang et al., 2022), the question arises whether this increase reflects movement complexity or is rather a consequence of intensified metabolic processes.

Seven studies further compared the effects of different movements, of which five^abdef^ studies reported differences in alpha activity. However, this extended to different parts of the cortex and thus does not show uniform activation in a specific area. Only three studies reported differences in the occipital cortex (e.g., Becker et al., 2023), which is associated with both visual processes (Zschocke et al., 2012) and working memory (Tuladhar et al., 2007). These differences could indicate that different types of movements place different demands on visual memory or working memory. More differentiated studies will have to single out these interwoven questions.

Four studies reported no exercise-induced differences (e.g., Chu et al., 2018), whereas Becker et al. (2023) reported a decrease in alpha activity during a movement-based exergame. This exercise was performed, analogous to the virtual condition of Baumeister et al. (2010), in the context of an exergame and effects resulting from the digital visual and acoustic presentation should not be neglected (Anders et al., 2018; Müller et al., 2023).

In summary, many studies reported alpha increases. These were primarily identified in the frontal, central, and parietal areas. The increases in the frontal and central areas are consistent with findings from previous EEG research involving predominantly cyclical endurance-based exercises. The parietal increase could also be a response to coordinative demands in connection with visual–spatial orientation, as these were also found in cognitive tasks that specifically required visual–spatial attention, internal attention, and intersensory reorientation (Benedek et al., 2014; Misselhorn et al., 2019; van Schouwenburg et al., 2017). In the context of dragon boat racing, Wu et al. (2023) showed no different activation between novices and experts. Baumeister et al. (2008), however, was able to demonstrate a difference in alpha activity between experts and novices in non-cyclical movements, which supports the relevance of differentiating between cyclical and non-cyclical movements. Even though alpha activity is increasingly shown in the complex movements investigated here, the coordinative demand cannot be considered solely responsible for the increase. An increase in alpha activity due to increased metabolism may also have been a contributing factor. However, there are tendencies suggesting that the coordinative aspect has an increasing effect, which is shown in an additional parietal increase. These activities occur at varying intensities and exercise durations.

### 4.4 Effects on beta frequency band

Only four studies reported an increase in beta activity triggered by sport (pre-post design = 4), five found no changes, and one study showed a decrease in beta activity (beta-1 in experts, Wu et al., 2023). The increases were shown only sporadically in the temporal, central, and frontal regions, but more consistently in the parietal and occipital cortex (*n* = 3; e.g. Rydzik et al., 2024). Among the studies that reported increases, exercises with greater strength or speed participation as well as increased endurance requirements were represented.

Beta activity is often associated with visual perception (Piantoni et al., 2010) or working memory (Deiber et al., 2007). It is important to note that this assignment is based on working memory according to Baddeley and Hitch (1974), which is related to sequential, visuospatial tasks and should not be generalized to proprioceptive, kinesthetic, and tactile tasks (Baddeley, 2018). Beta oscillations are often observed in various cortical areas, including the occipital (visual attention; Gola et al., 2013) or parietal area (visuospatial working memory; Deiber et al., 2007). In the context of movement execution, beta is increasingly reported in sensorimotor processes during fine motor movements (Engel and Fries, 2010; Lalo et al., 2007). Three of the four studies, except the one by Wind et al. (2020), which observed increased beta activity, examined exercises characterized by high conditional intensity (e.g., Wu et al., 2023). Such increased intensities may have been reflected in the elevated beta activity (Ciria et al., 2019; Hottenrott et al., 2013). However, it is difficult to separate sensorimotor control and attention, especially in sports, as sensorimotor behavior is often accompanied by various attentional processes (Engel and Fries, 2010). Therefore, the increases found in four studies may be the result of sensorimotor processes, increased attentional demands, or working memory processes. These effects have been shown primarily in the occipital and parietal areas, likely due to the specific cognitive demands, but less pronounced in the central areas. The assumption that the activation of beta oscillations is not solely the result of sensorimotor processes is strengthened by the phenomenon that beta increases were shown in areas that are not only associated with sensorimotor processes (e.g., Rydzik et al., 2024).

Five^adefh^ of the studies compared beta activity between different movements. One study on weightlifting acquisition showed a different activation in the right temporal area when comparing a movement performed according to different learning approaches (Ammar et al., 2024). The four remaining studies showed no difference in this frequency band.

Five studies reported no changes after or during exercise, and one indicated a decrease in beta-1 activity in the occipital region (experts, Wu et al., 2023). It should be noted that five of the nine studies did not differentiate between sub-bands of beta activity. Given the large range of 13-30 Hz, it is possible that this may have led to missing differences.

In sum, the findings for beta activity are not consistent: four studies found increases, five showed no changes, and one reported a decrease. Increases were primarily observed in the occipital and parietal cortex and may be attributed to increased demands on somatosensory processes, working memory, or visual processes (Deiber et al., 2007; Engel and Fries, 2010; Gola et al., 2013; Lalo et al., 2007). Our results differ from previous reviews with healthy subjects, which have reported frequent increases (Hosang et al., 2022). Whether the differences are caused by different levels of increased metabolism going along with endurance tasks needs further research. However, this difference could also be due to the predominantly shorter exercise duration, the lower average exercise intensity, or the increased coordinative demands within the exercises.

### 4.5 Effects on gamma frequency band

Gamma oscillations were examined in only six studies. Two reported increases (pre-post design = 2), two found no changes, and two showed a decrease. While increases were shown in the central area (Ammar et al., 2024; Henz et al., 2018), decreases were reported in the occipital and frontal cortex (Chu et al., 2018; Wind et al., 2020). Gamma activity is associated with movement control (Ball et al., 2008; Ulloa, 2022), attention (Jensen et al., 2007), and working memory processes (Thompson et al., 2021). Nevertheless, it is unclear whether gamma oscillations implement causal mechanisms of specific brain functions or represent a dynamic mode of neural circuit function. Therefore, Fernandes et al. (2017) speculated that gamma frequencies do not represent cognitive activity, but rather an activity motif that describes processes underlying information processing in brain circuitry. The results presented here are in line with previous studies on cyclic endurance tasks (Gramkow et al., 2020; Hosang et al., 2022), which also found inconsistent results regarding gamma activity. Both the increases and decreases could be the result of symbiotic effects of cognitive, somatosensory, and motor processes, as gamma effects have been shown at least in the first two contexts (Ball et al., 2008; Thompson et al., 2021; Ulloa, 2022).

Four^adef^ of the studies also compared gamma activation between different exercises or after varying executions of the movement. Only two studies (Ammar et al., 2024; Henz et al., 2018) reported different activations in the context of movement learning models, which, however, did not consistently emerge within any particular brain region.

In summary, no consistent trend in gamma activity could be identified. Similar to the delta frequency band, gamma has been investigated in only a few studies and show inconsistent results. Nevertheless, analyzing these oscillations in future investigations would be beneficial, since, on the one hand, contrary to previous assumptions, the delta frequency in particular is increasingly associated with cognitive processes (Harmony, 2013) and, on the other hand, a basis should be created to enable the analysis of potential interaction effects between the frequency bands (Varela et al., 2001).

### 4.6 Heterogeneity of exercises

The analyzed studies provide a scoping review of the current state of research on parallel complex movements with increased mental demands that have been examined using EEG. However, it should be noted that the cognitive demands of the individual movements vary considerably. While some exercises were influenced by external stimuli (Becker et al., 2023; Ben-Soussan et al., 2013; Visser et al., 2022), which required additional integration of external information, other movements, implemented within different learning models, were characterized by a high degree of variability in movement execution (Ammar et al., 2024; Henz et al., 2018; Henz and Schöllhorn, 2016; John and Schöllhorn, 2018). The study by Becker et al. (2023) was embedded in a research question to investigate movement tasks with executive control requirements. Therefore, the study’s objective is relevant for interpreting the findings, since it affected which brain areas and frequency bands were analyzed. The full range of cognitive processes required to successfully perform each movement is not yet fully understood. Despite this heterogeneity in mental demands, a trend has emerged indicating that alpha and theta activity can be increased through complex movements. However, this trend in theta activity did not emerge in studies investigating running and cycling at different intensity levels, which showed comparable quality in terms of EEG data acquisition and processing (Gramkow et al., 2020; Hosang et al., 2022). Whether these increases reflect cross-sport attentional and executive processes or rather sport-specific cognitive demands remains unclear. Additionally, effects resulting from elevated metabolic activity cannot be ruled out. Since EEG frequency bands have primarily been associated with purely cognitive tasks and mainly in the context of fine motor movements in sitting situations, their interpretation has thus far been largely limited to this context (Klimesch et al., 1998; Sauseng et al., 2007; Sauseng et al., 2005). So far, only a few studies in sport-related contexts indicate a stronger influence of coordinative demands on specific EEG frequency bands (Hülsdünker et al., 2020; Peterson and Ferris, 2018; Sipp et al., 2013). This connection is still very limited in EEG research and requires further investigation. In this context, further research is needed to eventually find clusters of movements that share comparable coordinative demands and similar influence EEG activity. This would allow for more precise identification and differentiation of sport-specific influences on EEG activity and would lead to a more differentiated application in the context of therapies.

### 4.7 Heterogeneity of EEG measurements

The studies investigated within this scoping review show differences in terms of EEG measurement and data processing. The modified criteria by Parr et al. (2021) for the assessment of EEG analyses and the associated effects on study quality (overall moderate study quality; *M* = 2.38) reveal the importance of the measurement and especially of correct preprocessing steps. EEG is an excellent device for recording brain activity, which is, however, contaminated by various external sources. Advanced denoising techniques, such as independent component analysis (ICA), play an important role in this process. Their primary aim is to distinguish brain activity from externally induced activity, such as muscle activity (Albera et al., 2012). Among the 13 studies, only seven studies (e.g., Visser et al., 2022) reported using advanced denoising techniques. This is problematic because, in the absence of the investigator’s expertise, the frequently reported frontal activity could be contaminated by eye movements or, due to continuous data collection, muscle activity may not be adequately detected and removed manually. Of the four studies that measured EEG during movement, where the separation of movement artifacts is particularly important, only two (Becker et al., 2023; Visser et al., 2022) used techniques such as ICA. This complicates the interpretation of the remaining two studies, as the removal of artifacts from the data cannot be transparently traced. In the study by Chu et al. (2018), preprocessing was carried out automatically by a ThinkGear™ chip, making it impossible to trace or evaluate the processing steps.

Only five studies (see Figure 2) differentiated alpha activity into alpha-1 and alpha-2, and four differentiated beta activity into beta-1 and beta-2. Greater differentiation would have been beneficial for two reasons. Firstly, a differentiated analysis would have identified potential differences in the sub-bands, and secondly, the relationship between cause and effect could have been demonstrated more effectively, as the sub-bands are typically associated with different functions. Moreover, the number of electrodes significantly impacts the quality of the data, which means that the quality generally increases with the number of electrodes (Lau et al., 2012). A higher number of electrodes not only facilitates source localization but also improves the separation of brain activity and artifacts through ICA, as more electrodes yield a greater number of independent components (Michel and Brunet, 2019). The average number of electrodes used in the studies was 20, which is sufficient to record EEG data of good quality (Miraglia et al., 2021). However, two studies utilized a limited number of electrodes. Chu et al. (2018) used only one, and Wollseiffen et al. (2016) used three electrodes for their measurements. The fact that none of the studies controlled for volume conduction further complicates the spatial interpretation of the locally measured activity (Parr et al., 2021; Rutkove, 2007). This is one of the main reasons for the overall moderate study quality and might be responsible for the inconsistent results across some frequency bands. In addition, study designs with mixed genders as well as with athletes and non-athletes were included in this review, which may have further contributed to the differing results (Corsi-Cabrera et al., 1993; Fang et al., 2022). The different methods of EEG measurement and preprocessing used in the respective studies make comparison difficult. Therefore, it is recommended to reach a common consensus regarding EEG frequency bands, EEG measurements, and especially the preprocessing of EEG data. The fact that both the posture of the body (Chang et al., 2011; Jung et al., 2020) and the location of measurement (indoor/outdoor; Boere et al., 2023) can have an influence on the measurement must also be taken into account. The heterogeneity resulting from the different methods is one of the reasons why no quantitative synthesis was conducted in this study.

## 5 Limitations and future research

In general, the limitations of the study are given by the boundary conditions of the study design and therefore do not allow for generalization or claim to be comprehensive. Although this review indicates that neural effects may be attributable to coordinative demands, this cannot be definitively concluded, as they may also have been increased as a result of metabolic activity (John et al., 2020). The absence of heart rate measurements in some studies further complicates the assessment of exercise intensity and, consequently, the evaluation of metabolic processes. Another aspect is the considerable heterogeneity of the analyzed movements in terms of their complexity. This is reflected not only in the varying cognitive demands, but also in the fact that some non-cyclic movements included pauses, for which no detailed information was provided in certain cases. In this review, brain activity was measured both during and after exercise and was mostly compared to activity before exercise. Even if measurements were taken immediately after the movement, the effect may already have subsided. In addition, Baumeister et al. (2010) only reported a comparison between activity during and after the movement, making it difficult to draw clear comparisons to pre-movement activity.

The years of publication reveal the growing relevance of the topic. Nevertheless, the number of studies is still limited, making it difficult to classify the effects of complex movements on EEG activity. In the context of movement-related EEG studies, it would be helpful for systematic findings to differentiate between changes in brain activation associated with cognitive demands, such as those arising from mathematical or language tasks, which are very often used in dual task paradigms (e.g., counting while walking); brain activation caused by increased metabolism (e.g., graded exercise test on a cycle ergometer, Gutmann et al., 2018), most often associated with an increase in heart rate; or changes in brain activation caused by an increase in complexity, resulting in increased information that has to be processed in parallel. In this context, it seems essential to specify the influence of sport-specific movements on EEG activity by analyzing complex movement forms in terms of their respective motor demands. This allows for a stronger integration of sport science explanations and reduces reliance on purely psychological models. It remains unclear whether the number of degrees of freedom involved in the movement has an influence on an EEG band. Future research could investigate other movements or games with different aims. The study by Visser et al. (2022) has shown that measurements taken during an open-skill exercise also provide usable information. In this context, it would be interesting to examine the neural effects of increased difficulty within an open-skill exercise, for example by increasing the pace of play. The influences of both increased intensity and increased coordinative demand would be of interest here. At the same time, the influence of psychological parameters occurring in parallel with complex movements must be examined to support generalization beyond controlled laboratory settings. Measurements immediately after the exercise are also valuable in the field of cognitive learning research (Sibley and Etnier, 2003; Tomporowski, 2003) as they enable the investigation of exercises in which measurements during movement would not have been possible due to the susceptibility of the EEG. As this review only examined exercises lasting a maximum of 20 min, it raises the question of how the effects might differ for exercises lasting an hour or more. How do frequencies, with a particular focus on frontal and central theta, behave over the course of an exercise or within an exercise with continuously increasing mental demands? How do pauses between movements influence neural activity? In this research context, potential moderator variables should be considered.

In general, a more holistic consideration of the integrative function should provide a more differentiated analysis of brain functions in the context of the diversity of movements in the future. With a focus on the field of sport and movement, it would help to differentiate the entire subjective information to be processed by the brain and to analyze its interdependencies. In addition to the multitude of coordinative loads to be performed in parallel, it would be necessary to differentiate between the different intensities of endurance and strength performance, the proportion of sensory systems involved and, in the case of team sports, the number of players, tactical and strategic tasks. This list could be expanded by all psychological parameters of influence.

## 6 Conclusion

Technical innovations combined with a shift in the focus of movements have led to an increased investigation of complex whole-body movements with many parallel actions. Based on the main findings of this scoping review, which indicate a trend toward increased theta and alpha activity, particularly in frontal, central, and parietal areas, the results support further investigation of complex movements. Since the number of studies is still limited and the heterogeneity of the complex movements examined here is high, more investigations are required to explore the potential influence of parallel complexity in more detail. Based on a consistent methodological EEG approach, future studies should consider the complexity of a movement and the resulting coordinative demands as potential moderator variables. In view of the beneficial role of the lower frequencies in general learning and therapeutic processes stimulated by complex movements, the extent of the consequences is only alluded to here, which nevertheless appears sufficient to intensify future research in this direction.

## Data Availability

The original contributions presented in the study are included in the article/Supplementary material, further inquiries can be directed to the corresponding author.

## Footnotes

^1^As this review addressed a different question, one component of the assessment tool by Parr et al. (2021), namely ‘secondary measures of verbal and conscious processing’ was removed as it was not relevant for our purposes.

^2^The original component ‘alpha definition’ from Parr et al. (2021) was modified to ‘brain wave definition’ similar to Hosang et al. (2022) because all EEG frequencies were of interest.

^3^In this section, the abbreviation in parentheses indicates the exercise that exhibits a higher EEG value in comparison.
