# Supplementary material for: Effects of complex whole-body movements on EEG activity: a scoping review
Source: Front Psychol. 2025 Jul 31;16:1547022. doi: 10.3389/fpsyg.2025.1547022 (PMC12351650; doi:10.3389/fpsyg.2025.1547022)
Supplement: Supplementary file 1 [file Table_1.docx]

**Supplementary Table 1.** Assessment of study quality based on QATQS (National Collaborating Centre for Methods and Tools, 2008) and Parr et al. (2021).

| **Study/ author** | **Selection bias** | **Study Design** | **Confounders** | **Blinding** | **Data collection method** | **Withdrawals and dropouts** | **Artifact handling** | **Definition of brain wave** | **Regional specificity** | **Temporal precision** | **Controls to volume conduction** | **Total** |
| --- | --- | --- | --- | --- | --- | --- | --- | --- | --- | --- | --- | --- |
| Ammar et al. 2024 | 2 | 1 | 2 | 2 | 3 | 1 | 1 | 3 | 1 | 2 | 3 | **2** |
| Baumeister et al. 2010 | 2 | 1 | 2 | 3 | 3 | 1 | 2 | 2 | 3 | 1 | 3 | **3** |
| Becker et al. 2023 | 2 | 1 | 1 | 3 | 2 | 1 | 1 | 2 | 1 | 1 | 3 | **2** |
| Ben-Soussan et al. 2013 | 2 | 1 | 3 | 3 | 3 | 1 | 2 | 3 | 2 | 2 | 3 | **3** |
| Chu et al. 2018 | 2 | 2 | 3 | 3 | 3 | 1 | 3 | 3 | 3 | 1 | 3 | **3** |
| Henz & Schöllhorn 2016 | 2 | 1 | 2 | 2 | 3 | 1 | 2 | 3 | 1 | 2 | 3 | **2** |
| Henz et al. 2018 | 2 | 1 | 2 | 2 | 3 | 1 | 1 | 3 | 1 | 2 | 3 | **2** |
| John & Schöllhorn 2018 | 2 | 1 | 1 | 3 | 2 | 1 | 1 | 2 | 1 | 2 | 3 | **2** |
| Rydzik et al. 2024 | 2 | 1 | 2 | 3 | 3 | 1 | 3 | 2 | 2 | 2 | 3 | **3** |
| Visser et al. 2022 | 2 | 1 | 1 | 3 | 2 | 1 | 1 | 2 | 1 | 1 | 3 | **2** |
| Wind et al. 2020 | 2 | 1 | 2 | 3 | 2 | 1 | 2 | 3 | 1 | 2 | 2 | **2** |
| Wollseiffen et al. 2016 | 2 | 1 | 2 | 3 | 3 | 1 | 2 | 2 | 3 | 2 | 3 | **3** |
| Wu et al. 2023 | 2 | 2 | 2 | 3 | 2 | 1 | 1 | 2 | 2 | 2 | 3 | **2** |

**References**

National Collaborating Centre for Methods and Tools, 2008. Qual. Assess. Tool Quant. Stud. URL https://www.nccmt.ca/knowledge-repositories/search/14 (accessed 5.15.24).

Parr, J.V.V., Gallicchio, G., Wood, G., 2021. EEG correlates of verbal and conscious processing of motor control in sport and human movement: a systematic review. Int. Rev. Sport Exerc. Psychol. 16, 396–427. https://doi.org/10.1080/1750984X.2021.1878548
